# Systematic Analysis of Tobacco CrRLK1L Family Genes and Functional Identification of *NtCrRLK1L47* in Environmental Stresses

**DOI:** 10.3389/fpls.2022.838857

**Published:** 2022-06-17

**Authors:** Xiaoxu Li, Cun Guo, Qi Wang, Zhiyuan Li, Jun Cai, Dousheng Wu, Yangyang Li, Aiguo Yang, Yongfeng Guo, Junping Gao, Liuying Wen, Wenxuan Pu

**Affiliations:** ^1^Technology Center, China Tobacco Hunan Industrial Co., Ltd., Changsha, China; ^2^Key Laboratory for Tobacco Gene Resources, Tobacco Research Institute, Chinese Academy of Agricultural Sciences, Qingdao, China; ^3^Hunan Key Laboratory of Plant Functional Genomics and Developmental Regulation, College of Biology, Hunan University, Changsha, China; ^4^Hunan Tobacco Research Institute, Changsha, China

**Keywords:** FERONIA receptor kinase, CrRLK1L family, environmental stresses, *NtCrRLK1L47*, tobacco

## Abstract

The *Catharanthus roseus* RLK1-like (CrRLK1L) family is involved in the regulation of plant reproduction, growth and development, cell wall integrity sensing, as well as responses to both biotic and abiotic stress conditions. Extraordinary progress has been made in elucidating the CrRLK1L family receptor kinases–mediated signaling pathway, while limited research addressed the functions of CrRLK1L proteins in tobacco. In this study, we identified and analyzed 48 NtCrRLK1L members from the tobacco genome. The newly identified NtCrRLK1L members were divided into seven groups together with the *Arabidopsis* CrRLK1L members. The syntenic analysis revealed that four pairs of *NtCrRLK1L* genes were predicted to have arisen from segmental duplication events. Expression profiling showed that the *NtCrRLK1L* genes were expressed in various tissues, and most *NtCrRLK1L* genes were induced by salt and drought stress conditions. Notably, *NtCrRLK1L47* was upregulated under drought and salinity stresses, and the NtCrRLK1L47-GFP fusion protein was located in the cell membrane. Furthermore, overexpression of the *NtCrRLK1L47* gene enhanced the salt tolerance in tobacco seedlings.

## Introduction

In the plant kingdom, the individuals live in a complex environment and are constantly threatened by various stresses. Hence, perceiving external signals and responding accordingly are vital to development and survival. The receptor-like kinase (RLK) family is one of the largest families, which can sense extracellular signals and then activate intracellular signaling pathways (Morillo and Tax, 2006; Lindner et al., 2012; Dievart et al., 2020; Wang et al., 2020). Among them, CrRLK1L proteins are the subfamily of RLKs, and might probably be unique to plants (Han et al., 2011; Lindner et al., 2012). Since the first CrRLK1L protein was discovered from the *Catharanthus roseus* cell cultures (Schulze-Muth et al., 1996), the family members have been identified in many species (Niu et al., 2016; Kou et al., 2017; Wang et al., 2021). Generally, the CrRLK1L family members possess three typical domains, including the intercellular cytoplasmic kinase domain, transmembrane domain, and extracellular ligands-binding domain (Dievart et al., 2020). Previous studies have shown that the extracellular domain is highly similar to the glycoprotein in *Xenopus*, suggesting that the ligands of the CrRLK1L family member may be modified by glycosylation (Schallus et al., 2008; Boisson-Dernier et al., 2011; Takeda et al., 2014). The CrRLK1L family members exhibited diverse expression patterns by triggering different biological processes (Hématy and Höfte, 2008; Lindner et al., 2012). The rapid alkalinization factor (RALF) peptides were identified to serve as the ligands, which could be perceived by the CrRLK1L members (Lindner et al., 2012; Xiao et al., 2019; Zhang et al., 2020b).

The CrRLK1L family members were reported to be involved in a variety of processes, including plant growth and development (Haruta et al., 2014), hormone signaling (Guo et al., 2009), stress responses (Ye et al., 2017), reproduction (Nibau and Cheung, 2011), energy production (Li et al., 2016), and RNA metabolism (Wang et al., 2020).

In *Arabidopsis*, a total of 17 CrRLK1L members were identified, and most of them were studied in detail to reveal their functions (Feng et al., 2019; Duan et al., 2020). As the founding member, FERONIA (FER) was first isolated from the semi sterile, female gametophytic mutant (Huck et al., 2003). In addition, the *FERONIA* was widely expressed in all vegetative tissues and developmental stages, which indicates that its role is not limited to the regulation of fertilization (Stegmann et al., 2017). With the remarkable progress, the FER was reported to act as the receptor of RALF1, RALF17, RALF23, RALF32, and RALF33, and also regulate the plant development and biotic/abiotic stress responses (Yu et al., 2018; Ge et al., 2019a; Xiao et al., 2019). Notably, FER was found to inhibit the abscisic acid (ABA) signaling pathway through activation of ABA Insensitive 2 (ABI2) (Chen et al., 2016). Furthermore, FER could confer the JA signaling pathway by phosphorylating MYC2 and is involved in regulating plant immunity (Guo et al., 2018). Furthermore, the THESEUS1 (THE1) was reported to play a key role in cell wall synthesis, cell expansion, and cell wall integrity (Van Der Does et al., 2017; Gonneau et al., 2018). Besides, ANX1/2 and BUPS1/2 were shown to work as a heteromeric receptor complex to coordinate the maintenance of pollen tube integrity and sperm cell discharge (Franck et al., 2018; Feng et al., 2019; Ge et al., 2019b). In addition, MDS1, MDS2, MDS3, and MDS4 were reported to have redundant roles in the metal ion stress response (Richter et al., 2018). In other species, several CrRLK1L members are identified. OsFLR1 and OsFLR2 are necessary for maintaining architecture, fertility, and seed yield (Li et al., 2016; Pu et al., 2017). In apples, MdFERL1 and MdFERL6 were reported to influence fruit ripening (Jia et al., 2017). PbrCrRLK1L3 and PbrCrRLK1L26 could regulate the pollen tube rupture and growth in pears (Kou et al., 2017).

Tobacco is not only a considerable economic crop but also a well-studied model organism. However, the extreme environment and diseases have always been a potential threat to tobacco yield and quality. The CrRLK1L members have been identified in *Arabidopsis* (Shiu et al., 2004), rice (Shiu et al., 2004), soybeans (Wang et al., 2021), cotton (Niu et al., 2016), pear (Kou et al., 2017), and apple (Zuo et al., 2018). The study of CrRLK1L members in tobacco is found to be limited. Therefore, the identification of the tobacco CrRLK1L is of great significance for the research of tobacco development and in response to biotic/abiotic stresses. With access to the tobacco genome, a systematic analysis by integrating bioinformatics and molecular biology was performed in the current study. The clues provided in this study would present the foundation for a further study focusing on these tobacco CrRLK1L members.

## Materials and Methods

### Identification and Phylogenetic Analysis

The version 4.5 of the genome sequence annotations of tobacco (*Nicotiana tabacum* L.) was downloaded from the SGN database (https://solgenomics.net/). All of the reported *Arabidopsis* CrRLK1L full-length protein sequences (Zhang et al., 2020c) were obtained from The Arabidopsis Information Resource (TAIR, https://www.arabidopsis.org/), and these sequences were used as queries to perform a BLASTP search against the tobacco protein database (E-value < 0.01). Then, these resulting sequences were used as new queries to conduct a BLASTP search again, to avoid missing potential members. The remaining members were named by their physical locations on the chromosome/scaffold. The amino acid number, molecular weights, and isoelectric points were predicted by using the ProtParam online tool (Garg et al., 2016).

The multiple sequence alignment of the new NtCrRLK1Ls and reported AtCrRLK1Ls were performed by using MAFFT v5.3 under default settings (Katoh and Standley, 2013). Then, the neighbor-joining phylogenetic analysis was conducted by MAGE X based on the alignment of full-length protein sequences with the bootstrap method of 1,000 replicates, substitution with the Poisson model, and pairwise deletion (Kumar et al., 2018).

### Motif and Gene Structure Analysis

The CrRLK1L motifs were identified by MEME online toolbox (http://meme-suite.org/) (Bailey et al., 2015). The GSDS tool (http://gsds.cbi.pku.edu.cn/) was adopted to visualize exon–intron organizations of the tobacco *CrRLK1L* genes by comparing their coding sequences (CDSs) and genomic sequences (Hu et al., 2015).

### Chromosomal Localization Analysis

The chromosomal localization information of *NtCrRLK1L* genes was obtained from the SGN database and was illustrated by Perl. The segmental duplications of *NtCrRLK1L* genes were predicted by the MCScanX program and were visualized by Circos of TBtools (Chen et al., 2020). The synteny relationship of the orthologous genes from tobacco and other plant species (*Arabidopsis*, tomato, potato, grape, and rice) was identified by the Synteny Plotter of Tbtools (Chen et al., 2020). The non-synonymous (ka) and synonymous (ks) substitutions of each duplicated gene pair were calculated by DnaSP v5 (Rozas et al., 2017).

### Promoter Analysis of Tobacco *CrRLK1L* Genes

The 2,000 bp region upstream of the *NtCrRLK1L* genes start site was selected to perform the promoter analysis as previously described (Cao et al., 2016). These promoter sequences were extracted from the genome sequence database. Then, the PlantCARE database was adopted for the *cis*-elements investigation (Lescot et al., 2002). The results were visualized by R.

### Expression Profiling of *NtCrRLK1Ls*

The reported RNA-seq data of tobacco tissues (Edwards et al., 2017) were downloaded from the GEO database (accession number: GSE95717). Then, the expression data of *NtCrRLK1L* genes were extracted and transformed with log2 to normalize the raw data. The pHeatmap R package was adopted to illustrate Heatmap under the default parameters.

### Tobacco Plants Preparation and Treatment

Cultivated tobacco K326 was used to analyze the expression pattern of *NtCrRLK1L* genes in this study. The roots, stems, leaves, and flowers were collected when tobacco grew to the budding stage, and the samples were stored in the refrigerator at −80°C for later use. The tobacco seedlings were germinated in Murashige and Skoog (MS) solid medium after disinfection, then they were transferred to a liquid MS medium and adapted for 5 days. Based on the previous reference, the seedlings were treated with 150 mmol/L NaCl liquid MS medium or placed on absorbent filter paper for salt and drought treatment, respectively, and the samples were harvested at 0, 1, 3, or 6 h after treatment (Li et al., 2021). All the samples were frozen in liquid nitrogen and transferred to −80°C for later use. Triple biological replicates were performed for each sample.

### RNA Extraction and qRT-PCR

Ultrapure RNA Kit (Cwbiotech, Beijing, China) was used to extract the total RNA of each sample, and the cDNA was synthesized using Evo M-MLV Mix Kit with gDNA Clean for qPCR (Accurate Biotechnology, Changsha, China). The qRT-PCR was performed on Roche LightCyclerR 480 by using SYBR Green Premix Pro Taq HS qPCR Kit (Accurate Biotechnology, Changsha, China). The tobacco ribosomal protein-encoding gene *L25* (GenBank No. L18908) was used as a control (Li et al., 2021). Three independent experiments were carried out with three technical replicates, and the average value was taken for analysis based on the 2^−ΔΔCt^ method (Livak and Schmittgen, 2001). The primers used are listed in Supplementary Table 1.

### Subcellular Localization Analysis of *NtCrRLK1L47*

The coding sequence of *NtCrRLK1L47* and *AtCLV1* (as cell membrane control) excluding the termination codon was amplified by Phanta Max Master Mix (Vazyme, Nanjing, China). Then, these sequences and green fluorescent protein (GFP) fragments were inserted into the pCHF3 vector separately, which was driven by the CaMV35S promoter. Furthermore, the CDS of *AtCLV1* and yellow fluorescent protein (YFP) fragments were inserted into the pCHF3 vector to construct *35S:: AtCLV1-YFP* vector. The reconstructed vectors were confirmed by sequencing (Sangon Biotech, Shanghai, China). The recombinant constructs and control were transiently transformed and expressed in *Nicotiana benthamiana* leaf epidermal cells for 3 days. Then, the GFP and YFP fluorescence signals were captured using a confocal microscope (TCS-SP8 Leica, Wetzlar, Germany).

### Generation of Transgenic Tobacco Plants and Analysis of Salt Stress Tolerance

To construct the overexpression vector, the CDS of *NtCrRLK1L47* was amplified by PCR and inserted into the pCHF3 vector by infusion (Clontech, Bejing, China). The recombinant plasmid was transformed into tobacco leaves by the *Agrobacterium*-mediated method (Buschmann, 2016). The T0 generation plants were screened on the MS medium containing 50 mg/L kanamycin. At T1 generation, the separation ratio was detected on the kanamycin medium to identify the homozygous lines. For salt stress assays, two homozygous lines and wild-type (K326) seeds were surface-sterilized and germinated in a vertical MS medium for 7 days. The seedlings with the same growth stage were transferred to MS medium with 0 or 150 mM NaCl, respectively, to observe changes in root length. The significant difference analysis was calculated by SPSS v18.0 with the *t*-test.

### Statistical Analysis

Statistical significance was determined by conducting Student's *t*-test or one-way ANOVA using SPSS 23.0 software (SPSS).

## Results

### Identification of *NtCrRLK1L* Genes

The BLASTP searches were used to identify *NtCrRLK1L* genes. A total of 48 *NtCrRLK1L* genes were identified. Then, the 48 *NtCrRLK1L* genes were renamed from *NtCrRLK1L01* to *NtCrRLK1L48* according to their physical locations on each chromosome/scaffold. Characteristics of the NtCrRLK1L proteins, including the number of amino acids, molecular weight (MW), and isoelectric point (pI) were analyzed. The length of 48 NtCrRLK1L proteins ranged from 641 (NtCrRLK1L42) to 1,842 amino acids (NtCrRLK1L33). The molecular weight of NtCrRLK1L proteins ranged from 71.34 (NtCrRLK1L42) to 205.29 (NtCrRLK1L33) kDa, and isoelectric points ranged from 6.24 (NtCrRLK1L42) to 8.41 (NtCrRLK1L05). The detailed information could be explored in Supplementary Table 2.

### Multiple Sequence Alignment and Phylogenetic Analysis

**Figure 1:**
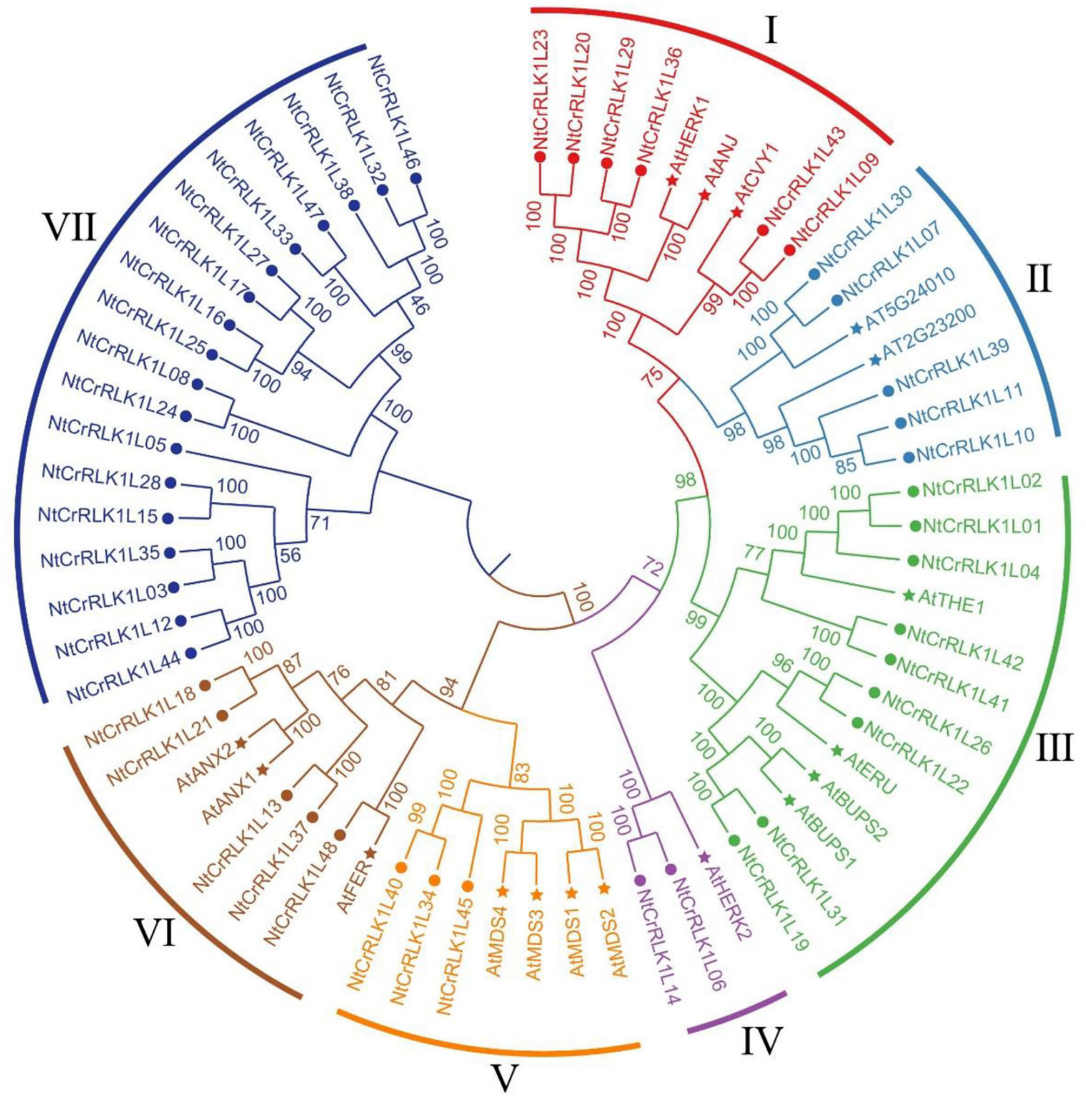
Phylogenetic analysis of tobacco NtCrRLK1L family members. The phylogenetic tree was generated from the alignment of the full-length protein sequence of CrRLK1L proteins from tobacco and *Arabidopsis* by the neighbor-joining (NJ) method. The tobacco CrRLK1L members together with their *Arabidopsis* homologs were classified into 7 groups, and distinct groups were marked by different colors.

To explore the phylogenetic relationship of the NtCrRLK1L proteins, the neighbor-joining tree was constructed based on the multiple sequence alignment of the tobacco CrRLK1L members and reported *Arabidopsis* members. As a result, those CrRLK1L members were classified into seven groups (Figure 1). Generally, most of the groups contained CrRLK1L members from tobacco and *Arabidopsis*, indicating that the expansion of CrRLK1L members might appear before the divergence of those species. Among the 7 groups, groups VII and IV contained the largest (18) and smallest (3) numbers of genes, respectively. In Group VI, only NtCrRLK1L48 was divided with AtFER, while AtTHE1 was clustered with multiple NtCrRLK1Ls in group III, which implied that the CrRLK1L members experienced duplication event after species differentiation. Intriguingly, it was found that only the tobacco CrRLK1L proteins fall in Group VII, indicating that the generation of these *NtCrRLK1L* genes might be caused by duplication events after the differentiation of tested species.

### Gene Structure and Conserved Motif Analysis

The GSDS was adopted to analyze the tobacco *CrRLK1L* gene structure. As a result, the intron number of studied genes was found to range from 0 to 28 (Supplementary Figure 2). Interestingly, 14 (29.17%) *NtCrRLK1L* genes were interrupted by one or two introns, whereas 15 (31.25%) *NtCrRLK1L* genes did not hold any introns. Furthermore, in Group VII, 18 *NtCrRLK1L* members contained an average of 18.5 introns, of which *NtCrRLK1L33* harbored the maximum number of introns, as high as 28 introns. In particular, the *AtCrRLK1L* members were also analyzed together with *NtCrRLK1L*, and the result showed that *CrRLK1L* genes from tobacco and *Arabidopsis* shared similar gene structures in the same group.

In addition, the CrRLK1L protein sequences were analyzed by the MEME toolbox. As a result, a total of 10 motifs were identified, namely, motif 1–10 (Supplementary Figure 1). As displayed in Supplementary Figure 2, the lengths of these conserved motifs ranged from 21 to 50 aa. Among them, motifs 1, 4, and 6 could be found in all studied CrRLK1L members. Inconsistent with the results of gene structure analysis, the CrRLK1L proteins in the same group usually have similar motifs' types and orders. Notably, the motif compositions of the Group VII members are not as consistent as the other group. For example, only 6 motifs were found in the protein sequence of NtCrRLK1L12 and NtCrRLK1L44, while in NtCrRLK1L33, there were 18 motifs detected. The similarities of characteristic motifs in each group may reflect functional similarities.

### *NtCrRLK1L* Genes Syntenic Analysis

**Figure 2:**
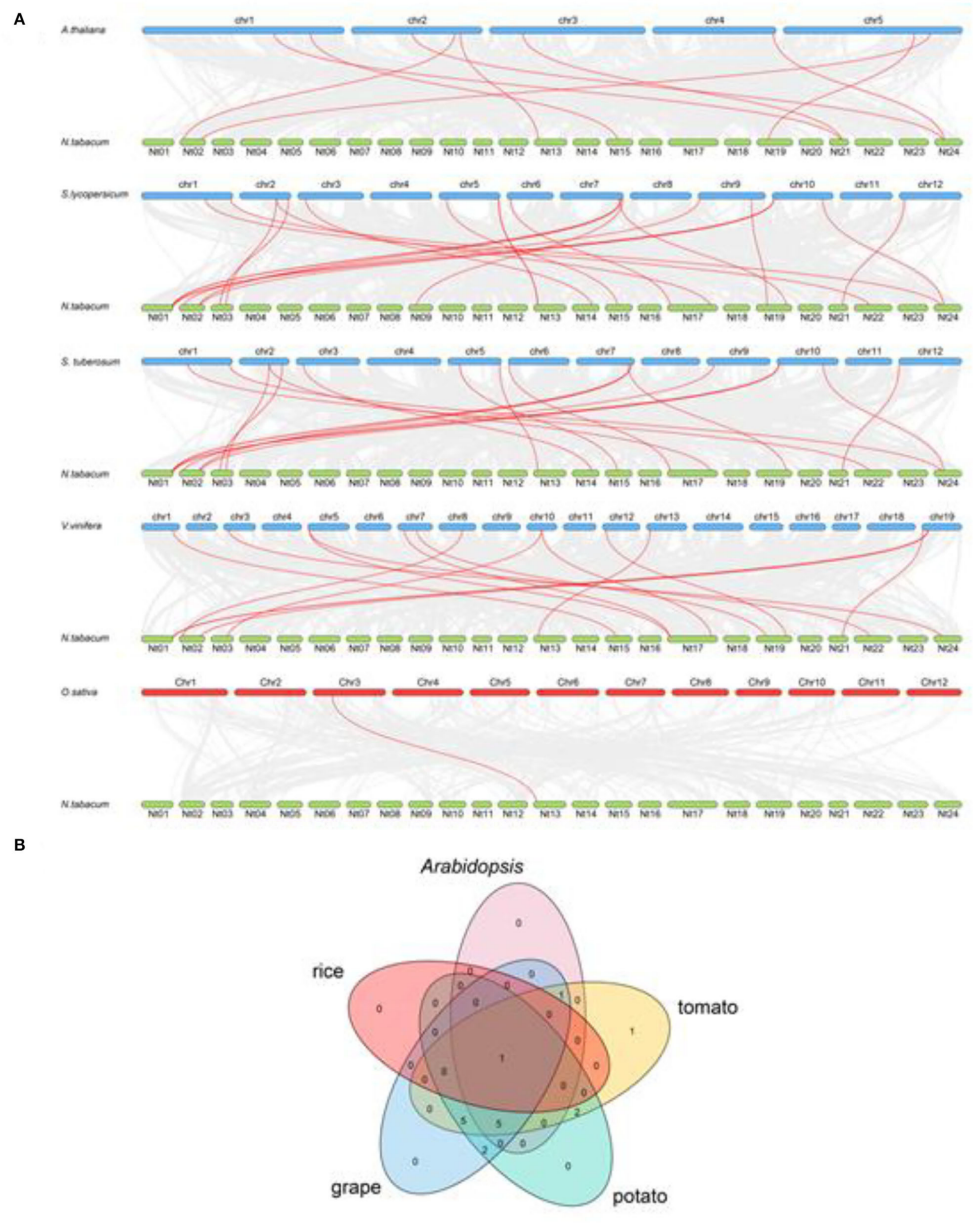
The synteny analysis of CrRLK1L members between tobacco and five other species. **(A)** The gray line represents all the collinear blocks between tobacco and five other species, while the syntenic *CrRLK1L* gene pairs were marked by the red line. **(B)** The CrRLK1L members formed the syntenic pairs between tobacco and five other species, which were analyzed and visualized by the Venn plot.

To further study the evolutionary relationship among the tobacco *CrRLK1L* genes, syntenic analysis was carried out between tobacco and five plant species, including four dicots (*Arabidopsis*, tomato, potato, and grape) and one monocot species (rice). The result showed that there was a syntenic relationship between 15 of the *NtCrRLK1L* genes with *CrRLK1L* genes in tomato, 14 *NtCrRLK1L* genes in grape, 7 *NtCrRLK1L* genes in *Arabidopsis*, 15 *NtCrRLK1L* genes in potato, and 1 *NtCrRLK1L* gene in rice. The numbers of predicted collinear pairs between tobacco and tomato, grape, *Arabidopsis*, potato, and rice were 17, 15, 9, 17, and 1, respectively (Figure 2A).

Particularly, *NtCrRLK1L09* were predicted to form collinear pairs with *CrRLK1L* genes from the five tested species, indicating that this gene may arise before the separation of these species (Figure 2B). Notably, a total of six collinear gene pairs were identified within tobacco and tomato/grape/*Arabidopsis*/potato members, indicating that those pairs may appear after the divergence of dicot and monocot species except *NtCrRLK1L09*.

### Chromosomal Distribution and Duplication Events

**Figure 3:**
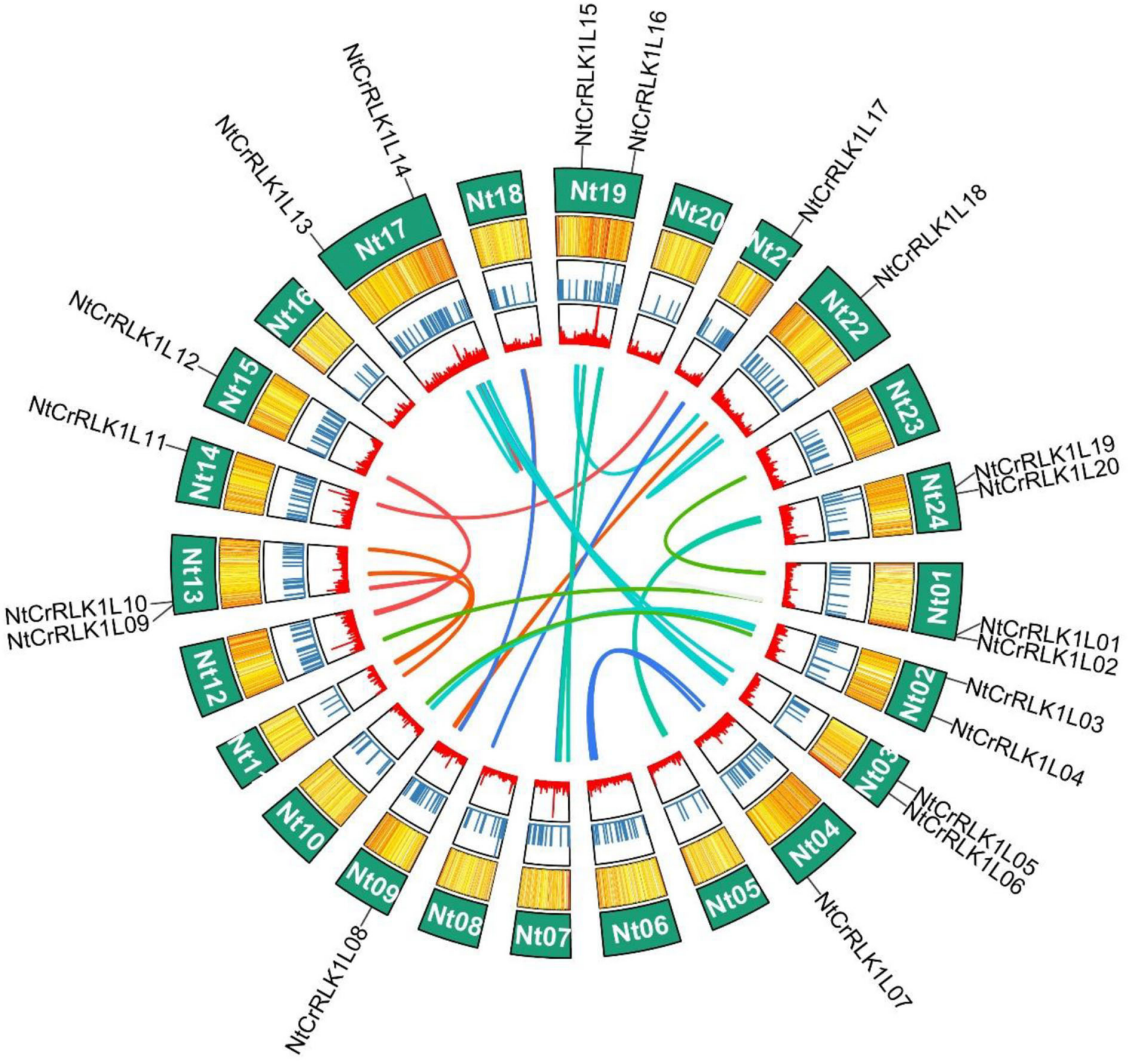
Distribution of *NtCrRLK1L* genes on tobacco chromosomes. In tobacco, 20 *CrRLK1L* genes were mapped to 13 tobacco chromosomes, the remaining members were located on scaffolds (not shown in this figure).

The chromosomal location information of newly identified *NtCrRLK1L* genes was obtained from the SGN database and visualized using the R. As a result, 20 of the 48 *NtCrRLK1L* genes were mapped onto 13 tobacco chromosomes (Figure 3), while the remaining *NtCrRLK1L* genes were located on scaffolds. There were two tobacco *CrRLK1L* genes found on chromosomes 1, 2, 3, 13, 17, 19, and 24, while chromosomes 4, 9, 14, 15, 21, and 22 only harbored one *CrRLK1L* gene. It has been reported that a chromosome region possessing two or more genes within 200 kb is defined as a cluster (Li et al., 2019). The location information showed that *NtCrRLK1L32* and *NtCrRLK1L33* formed one cluster on scaffold Nitab4.5_0005453 (Supplementary Table 2).

**Figure 4:**
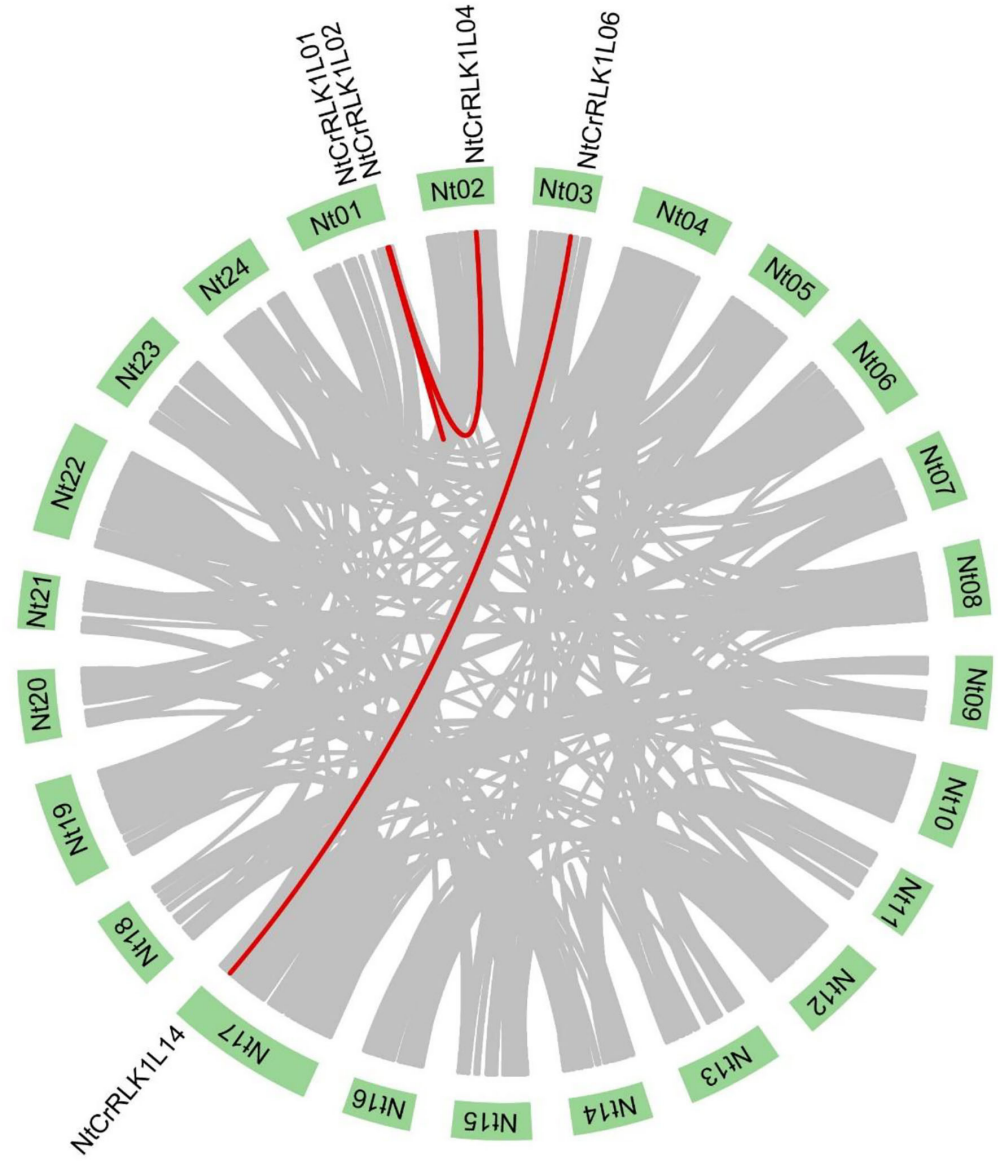
Segmental duplication analysis of the *NtCrRLK1L* genes. In the tobacco genome, all the putative segmental duplication pairs were marked by gray color, and the red lines represented the *NtCrRLK1L* segmental duplication pairs.

The segmental duplication was reported to play important role in the expansion of the gene family. In this study, the MCScanX was used to analyze the segmental duplication of NtCrRLK1L members. As a result, a total of five *NtCrRLK1L* genes were identified to form 4 segmental duplication pairs (Figure 4). As the reference, the ratio of non-synonymous (Ka) and synonymous (Ks) is one of the important parameters for evolution analysis (Cao et al., 2016). In the current study, the Ka/Ks values of 4 segmental duplication pairs were <1 (Supplementary Table 4), implying that those 4 *NtCrRLK1L* duplication pairs might have experienced the purifying selective pressure. On the other hand, this result also suggested that the purifying selection occurs in the evolution of the tobacco CrRLK1L family and might play an important role in the conservative function of *NtCrRLK1L* members.

### Analysis of *NtCrRLK1L* Gene Promoters

**Figure 5:**
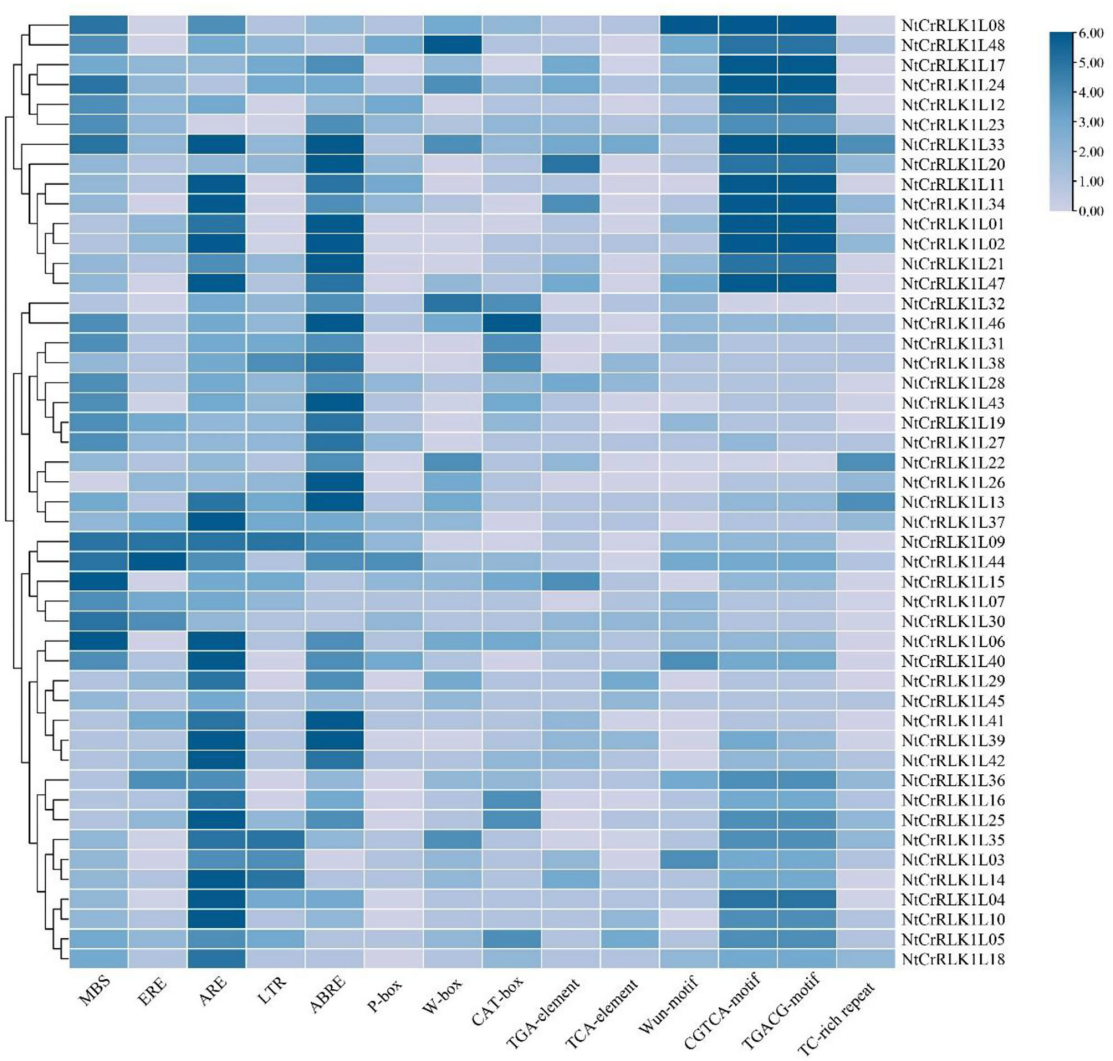
Statistics diagram of *cis*-elements in the promoter region of *NtCrRLK1L* genes. The color represents the numbers of *cis*-element in *NtCrRLK1L* gene promoters.

To study the regulatory features of the family genes globally, the promoter regions of 48 *NtCrRLK1L* genes were analyzed by the PlantCARE Online toolbox. Results showed that many *cis*-elements were detected in the promoter regions of *NtCrRLK1L* genes, and 14 *cis*-elements were selected from the PLACE database for visualization (Figure 5). The *cis*-elements related to stress responses including W-box, MBS, TC-rich repeats, Wun-motif, LTR, and ARE were detected in the promoter regions of many *NtCrRLK1L* genes, indicating that these genes might be involved in various stress responses. In addition, several hormone-response elements were also identified in promoter regions of *NtCrRLK1L* genes, including ABRE, TCA-element, ERE, and the CGTCA-motif. In particular, the observation of the ABRE element in the promoter regions of all *NtCrRLK1L* genes suggests that those genes might be involved in ABA-mediated stress responses.

### Expression Profile of *NtCrRLK1L* Genes

**Figure 6:**
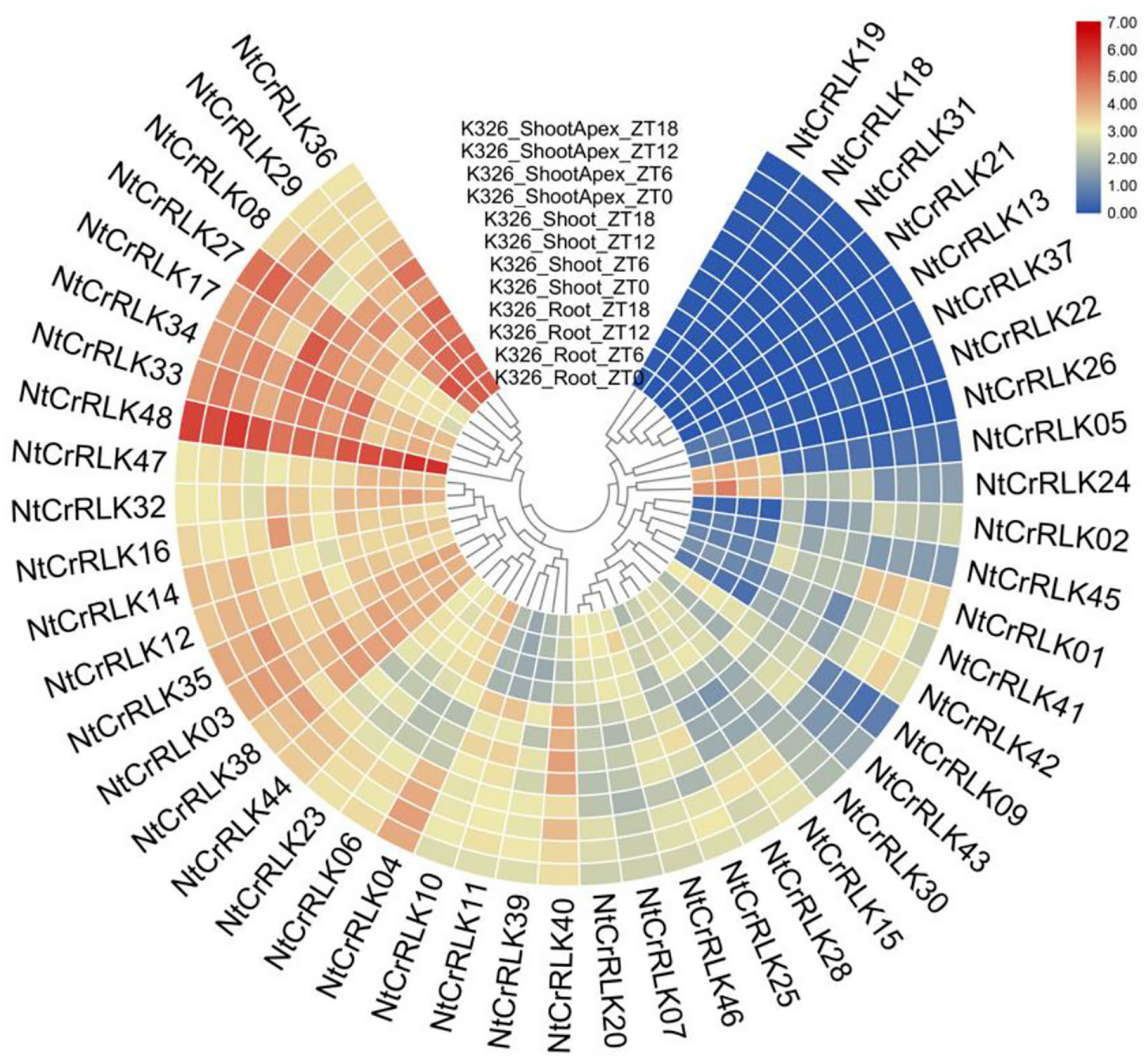
Expression patterns of the *NtCrRLK1L* genes in the tested tissues (root, shoot, and shoot apex). The data were retrieved from transcriptome data and visualized by R.

To preliminarily elucidate the roles of *NtCrRLK1L* genes in tobacco growth and development, the RNA-seq data about *NtCrRLK1L* genes in three different tissues were obtained from the NCBI database and analyzed (Edwards et al., 2017). As the result (Figure 6), among 48 *NtCrRLK1L* genes, 40 were detected to be expressed in at least one of the three tested tissues, and eight were highly expressed in all three tissues, especially *NtCrRLK1L48*. Furthermore, several genes exhibited tissue-specific expression patterns. For example, the expression of *NtCrRLK1L05* in Group VII was detected in the roots only. In Group III, *NtCrRLK1L02* and *NtCrRLK1L42* were detected exclusively in the roots. Similarly, *NtCrRLK1L09* was detected exclusively in the shoot apex. Notably, expressions of eight *NtCrRLK1L* genes were not detected in those three test tissues.

### Validation and Enrichment of Expression Patterns by qRT-PCR

**Figure 7:**
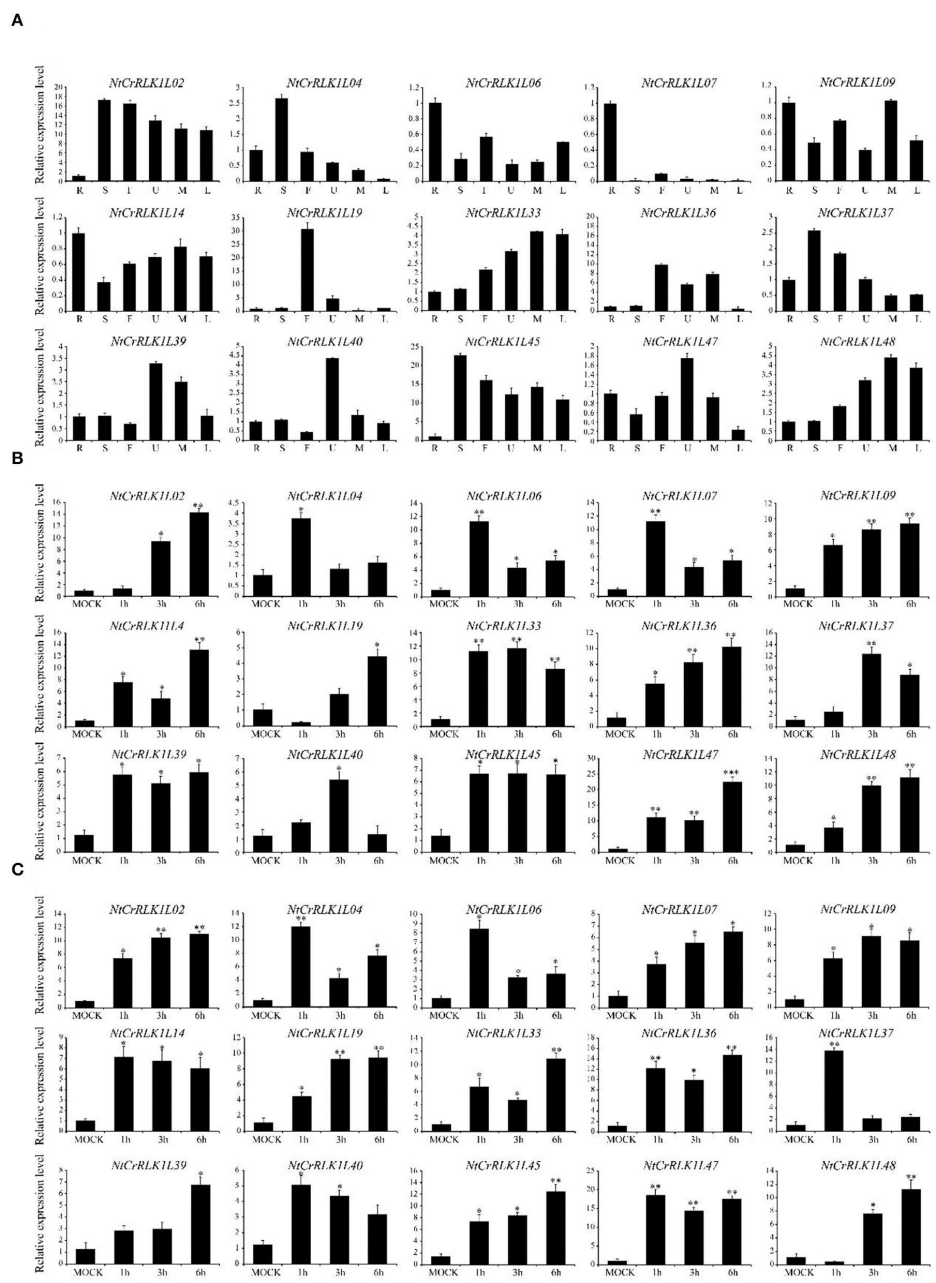
qRT-PCR analysis of representative *NtCrRLK1L* genes from different groups. **(A)** Expression patterns of the *NtCrRLK1L* genes in different tissues. R (root), S (stem), F (flower), U (upper leaf), M (middle leaf), and L (lower leaf). To verify the tissue specificity expression of the selected *NtCrRLK1L* genes, the expression level of each gene was calculated relative to the root tissue. **(B)** The expression level of the selected *NtCrRLK1L* genes under salt stress treatments. **(C)** The expression level of selected *NtCrRLK1L* genes under drought stress treatments. The data were means ±SD from three independent replications. *p < 0.05; **p < 0.01.

To confirm and enrich the expression patterns of the *NtCrRLK1L* genes from RNA-seq analysis, representative *NtCrRLK1L* genes from different groups were selected to perform qRT-PCR analysis. The tobacco tissues including root, stem, flower, upper leaf, middle leaf, and lower leaf were obtained to detect the expression changes of *NtCrRLK1L* genes. As a result (Figure 7), the selected *NtCrRLK1L* genes exhibit multiple expression patterns. For instance, *NtCrRLK1L02* was highly expressed in the other five tissues exclusively in the roots, and *NtCrRLK1L47* and *NtCrRLK1L48* were detected in all six tested tissues, which was consistent with the RNA-seq analysis. In Group II, *NtCrRLK1L07* exhibited a tissue-specific expression pattern and was expressed exclusively in roots, while *NtCrRLK1L39* was expressed in all tested tissues. This result suggested that the functional differentiation may occur in the same group of *NtCrRLK1L*.

Furthermore, several *NtCrRLK1L* genes were selected to test whether they could respond to salt and drought stresses. As a result, the expression patterns of most *NtCrRLK1L* genes under salt or drought stress were time-specific. For example, the expression level of *NtCrRLK1L04, NtCrRLK1L06*, and *NtCrRLK1L07* reached the peak at 1 h of salt treatment, then dropped sharply. Notably, *NtCrRLK1L19* was inhibited after 1 h of salt treatment, while its expression level reached a peak (~4-fold) after 6 h of salt treatment, and the expression of the *NtCrRLK1L19* gene continued to rise under drought treatment. Among them, *NtCrRLK1L47* had the highest expression level no matter the salt and drought treatment. Its expression level reached the peak after 6 h by the salt treatment (22-fold) and 1 h by drought treatment (18-fold). Thence, the *NtCrRLK1L47* gene was selected for further study.

### Subcellular Localization Analysis of *NtCrRLK1L47*

**Figure 8:**
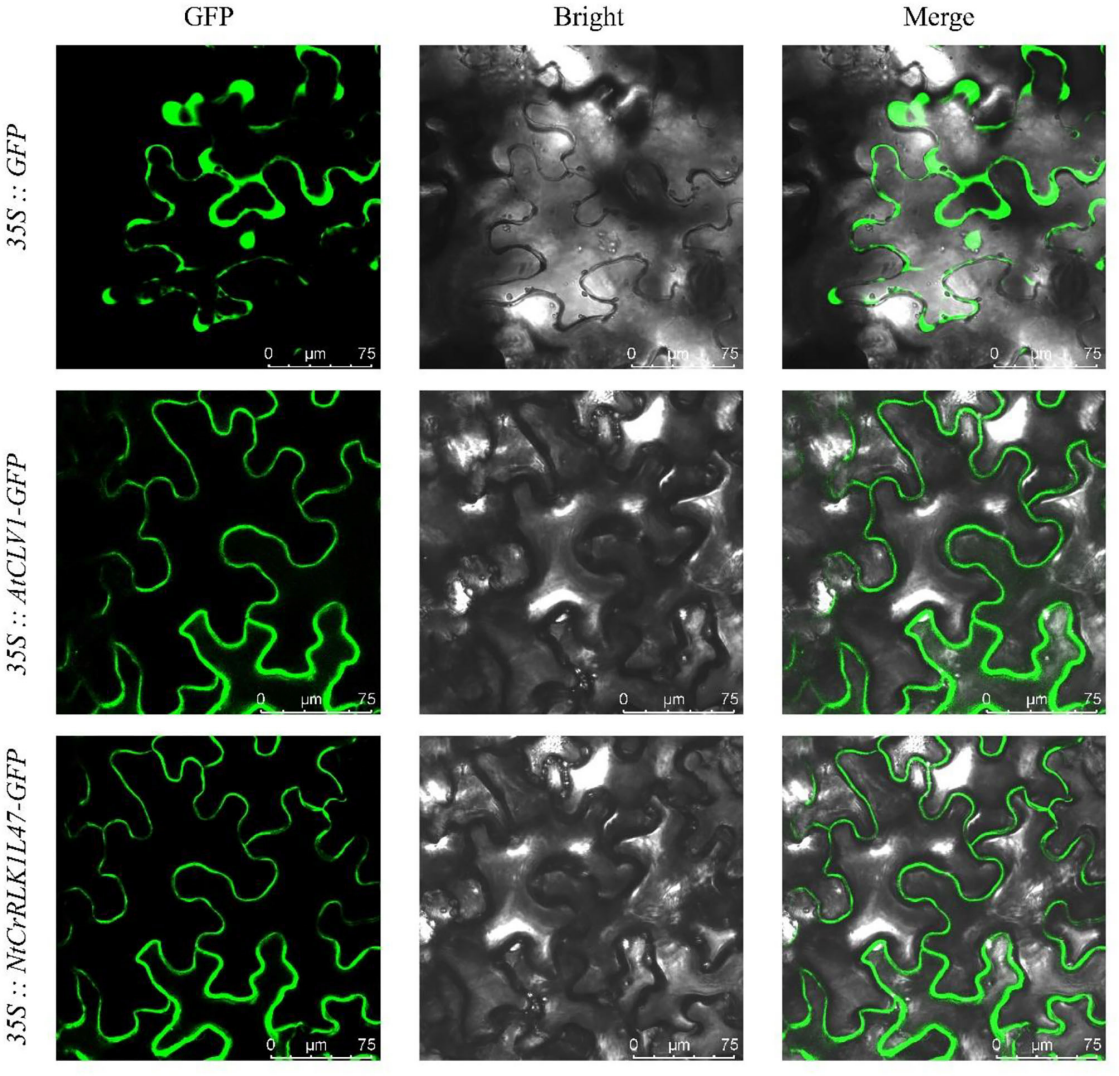
Subcellular localization of NtCrRLK1L47 protein within the cell by GFP assays. The *35::GFP, 35::AtCLV1-GFP*, and *35::NtCrRLK1L47-GFP* constructs were transiently transformed and expressed in *Nicotiana benthamiana* leaf epidermal cells; the GFP signals were visualized on a confocal laser microscope.

To further explore the subcellular localization of the *NtCrRLK1L* genes, the subcellular localization of *the NtCrRLK1L47* gene was selected for analysis (Figure 8). As revealed by confocal microscopy, the green fluorescence of the AtCLV1-GFP is only distributed on the cell membrane as previously reported (Stahl et al., 2013), whereas the signal of GFP protein was found to distribute throughout the whole cell. In addition, the signal of NtCrRLK1L47-GFP fusion protein was specifically confined to the membrane. Besides, the co-localization assay was carried out, and the fluorescence signal of the NtCrRLK1L47-GFP fusion protein was found to be consistent with AtCLV1-YFP (Supplementary Figure 3). Considering these clues, NtCrRLK1L47 protein was proved to localize on the membrane.

### Overexpression of *NtCrRLK1L47* Enhanced the Salt Tolerances

The genetic experiments were performed to explore the potential function of the *NtCrRLK1L47* genes in salt tolerance, and we obtained two overexpression lines of the *NtCrRLK1L47* gene, *NtCrRLK1L47*-*OE4* and *NtCrRLK1L47-OE5*. Then, the root elongation assay was carried out and no obvious differences were observed between wildtype and *NtCrRLK1L47-OE* lines under normal growth conditions. However, the *NtCrRLK1L47-OE4* and *NtCrRLK1L47-OE5* lines showed better growth performance than wildtype plants under salt treatments. The root elongation assay showed that significantly longer roots of the two independent overexpression lines were measured compared to the wildtype on 150 mM NaCl. This result indicated that *NtCrRLK1L47* conferred salt tolerance on transgenic tobacco seedlings.

## Discussion

The *CrRLK1L* gene family has been reported to play important roles in plant development, metabolism, and stress responses. In the current study, a comprehensive analysis of the CrRLK1L family in tobacco was carried out. As a result, a total of 48 tobacco CrRLK1L members were identified. Furthermore, the phylogenetic and syntenic analyses were integrated to identify the homologs of tobacco *CrRLK1L* genes with reported members and infer their potential functions. Furthermore, to verify these inferences, several genes were selected to perform the expression and function analysis.

In the current study, CrRLK1L family members of tobacco and *Arabidopsis* were clustered within seven groups. In Group III, NtCrRLK1L19 was clustered together with AtBUPS1 and AtBUPS2 (Figure 1), and their coding genes were predicted to form the collinear pairs (Figure 2), suggesting that the *NtCrRLK1L19* might be the homolog of *AtBUPS1* and *AtBUPS2*. Interestingly, it was indicated that the reactive oxygen species (ROS) served downstream of the BUPS/ANX receptor complex, which might confer stress response complex (Zhang et al., 2020a; Kim et al., 2021). Meanwhile, our results revealed that the promoter of the *NtCrRLK1L19* gene contains many stress-response elements, and this gene was proved to be induced by salt stress. These clues implied, as the homolog of *AtBUPS1* and *AtBUPS2*, the *NtCrRLK1L19* gene might be involved in the stress responses. Furthermore, in the same group, AtTHE1 was reported to function in plant cell wall synthesis and cell expansion and detected to express in root and shoot (Hématy et al., 2007; Merz et al., 2017). In our study, NtCrRLK1L01, NtCrRLK1L02, and NtCrRLK1L04 were clustered together with AtTHE1 (Figure 1), and their encoding genes were predicted to form collinear pairs with *AtTHE1*, respectively (Figure 2), proving that these tobacco genes might be homologous genes of *AtTHE1*. Furthermore, *NtCrRLK1L01, NtCrRLK1L02*, and *NtCrRLK1L04* were identified to form two segmental duplication pairs, implying that duplication events were raised after the segregation of these two species (Figure 4). Interestingly, the expression analysis showed that the expression patterns of these genes were discrepant. *NtCrRLK1L01* and *NtCrRLK1L02* were detected to mainly express in the shoot apex, while *NtCrRLK1L04* expressed in all detected tissues, especially more in the stem (Figures 6, 7). The results presented here indicated that these duplicated genes might undergo subfunctionalization.

In Group I, NtCrRLK1L09 was clustered with AtCVY1, and the encoding gene was predicted to form collinear pairs with *AtCVY1* and *OsFLR13*, respectively (Figures 1, 2). Interestingly, OsFLR13 was clustered with AtCVY1 in a previous study (Yang et al., 2020), which implies that *NtCrRLK1L09* acts as the homolog of *OsFLR13* and *AtCVY1* in tobacco. AtCVY1 was reported to regulate cell morphogenesis, flowering, and seed production. In the current study, *NtCrRLK1L09* was detected to express in flowers (Figure 7), suggesting this gene might undertake the conserved function in tobacco.

**Figure 9:**
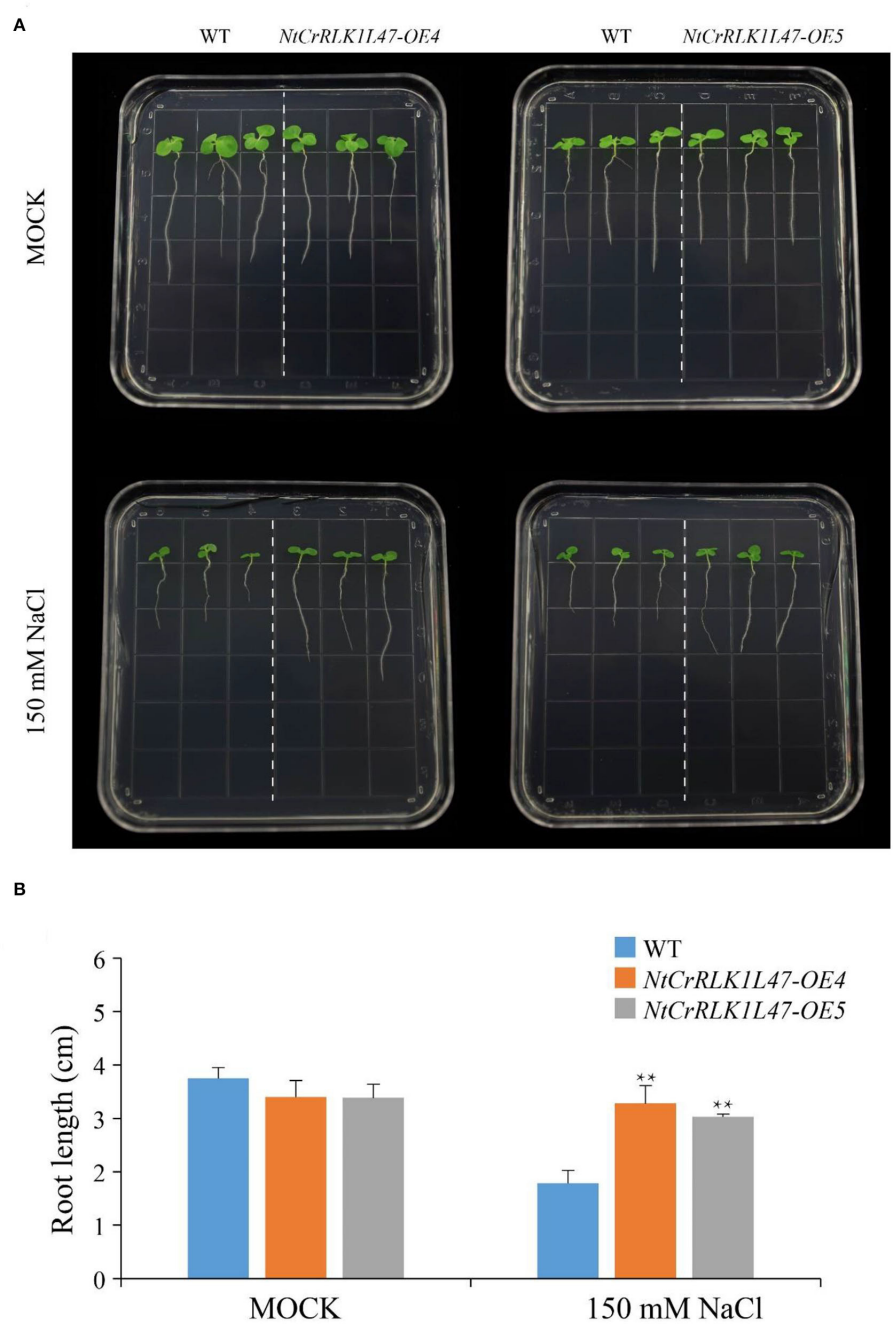
Phenotype and function analysis of *NtCrRLK1L47* gene in transgenic tobacco under salt stress conditions. **(A)** Root growth experiment of wild type and *NtCrRLK1L47* overexpression lines under 0 or 150 mM NaCl treatment. **(B)** The quantification of tobacco root length and the data were collected from more than 15 plants per genotype with three biological replicates. WT, wild type. Values represent means ± SD. **p < 0.01 (*t*-tests).

Furthermore, the *CrRLK1L* genes in Group VII were unique to tobacco (Figure 1), suggesting that this family might appear after the differentiation of these two species. In addition, *NtCrRLK1L32* and *NtCrRLK1L33* were found to fall into the same cluster (Supplementary Table 2), indicating that the duplication event might drive the expansion of this group. Considering the unsatisfactory quality of the tobacco genome, most members from this group could not map the chromosomes, and there might be chances to identify duplication events of this group in the future. Furthermore, various stress-response *cis*-elements were found to attend in the promoter region of this group members, including W-box, MBS, and ARE (Figure 5), indicating that the members of this group might be involved in stress responses. Then, several representative genes were investigated with the expression profile under drought and salt stresses (Figure 7). Notably, *NtCrRLK1L47* could be highly induced by these two treatments (Figure 7). The subcellular localization analysis showed that NtCrRLK1L47-GFP fusion protein was localized on the membrane (Figure 8). Not surprisingly, it was found that the overexpression of the *NtCrRLK1L47* gene could enhance the salt tolerance in tobacco seedlings (Figure 9), suggesting that NtCrRLK1L47 may act as a membrane receptor regulating stress responses.

## Conclusion

In this study, we identified 48 *CrRLK1L* genes in the tobacco genome, and comprehensive and systematic analyses were performed to interpret the evolutionary relationship of CrRLK1L family members. Furthermore, the promoter *cis*-element and expression pattern analysis showed that many *cis*-elements related to stress responses existed in the promoter regions of the *NtCrRLK1L47* gene, and *NtCrRLK1L47* was significantly up-regulated under salt stress conditions. Furthermore, the genetic experiments also confirmed that NtCrRLK1L47 provides effective resistance against salt stressors. These results provide insight into understanding the evolutionary mechanism of *NtCrRLK1L* genes and could be helpful to explore the biological functions of NtCrRLK1L members in the future.

## Data Availability Statement

The datasets presented in this study can be found in online repositories. The names of the repository/repositories and accession number(s) can be found in the article/Supplementary Material.

## Author Contributions

XL and CG conducted the research and drafted the manuscript. QW, ZL, JC, DW, YL, AY, YG, and JG assisted in data collection and analysis. LW and WP conceived the research and drafted the manuscript. All authors read and approved this final manuscript.

## Funding

This research was financially supported by the China Tobacco Genome Project [110202001029(JY-12) and 110202101003(JY-03)], the China Tobacco Hunan Industrial Co., Ltd. Technology Project (KY2021YC0001), and the Agricultural Science and Technology Innovation Program (ASTIP-TRIC01 & 02).

## Conflict of Interest

XL, JG, and WP were employed by China Tobacco Hunan Industrial Co., Ltd. The remaining authors declare that the research was conducted in the absence of any commercial or financial relationships that could be construed as a potential conflict of interest.

## Publisher's Note

All claims expressed in this article are solely those of the authors and do not necessarily represent those of their affiliated organizations, or those of the publisher, the editors and the reviewers. Any product that may be evaluated in this article, or claim that may be made by its manufacturer, is not guaranteed or endorsed by the publisher.

## Supplementary Material

Supplementary Table 1 qRT-PCR primers and sequences used in this study.

Supplementary Table 2 Features of NtCrRLK1L genes identified in tobacco.

Supplementary Table 3 Synteny blocks of *NtCrRLK1L* genes between tobacco and other five plant species.

Supplementary Table 4 The detailed information of segmental duplication of gene pairs within the tobacco genome.

Supplementary Figure 1 Conserved motifs and exon–intron structure organizations of the NtCrRLK1L members.

Supplementary Figure 2 The detailed sequence information for each motif in the NtCrRLK1L proteins.

Supplementary Figure 3 The co-localization assay of NtCrRLK1L47 with AtCLV1.

Supplementary Figure 4 The relative expression level of the *NtCrRLK1L47* gene in wild type and two overexpression lines; the relative expression level of each genotype was calculated relative to the wild type.
