# Supplementary figures and images for: Systematic Analysis of Tobacco CrRLK1L Family Genes and Functional Identification of NtCrRLK1L47 in Environmental Stresses
Source: Front Plant Sci. 2022 Jun 17;13:838857. doi: 10.3389/fpls.2022.838857 (PMC9247620; doi:10.3389/fpls.2022.838857)

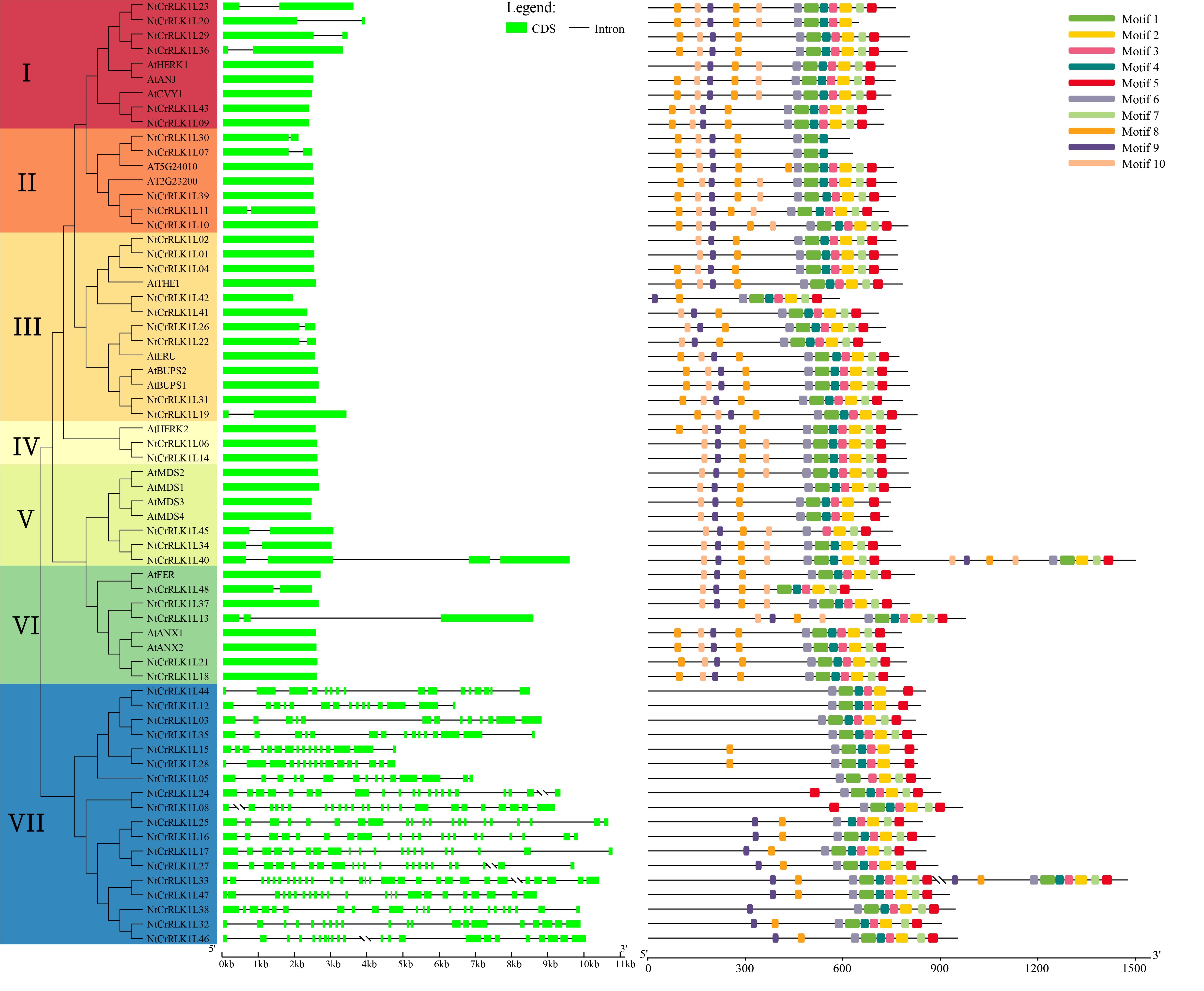

Supplement: Supplementary Table 1 — qRT-PCR primers and sequences used in this study. [file Data_Sheet_1.ZIP › Supplementary_Material/Supplementary Figure 1.jpg]

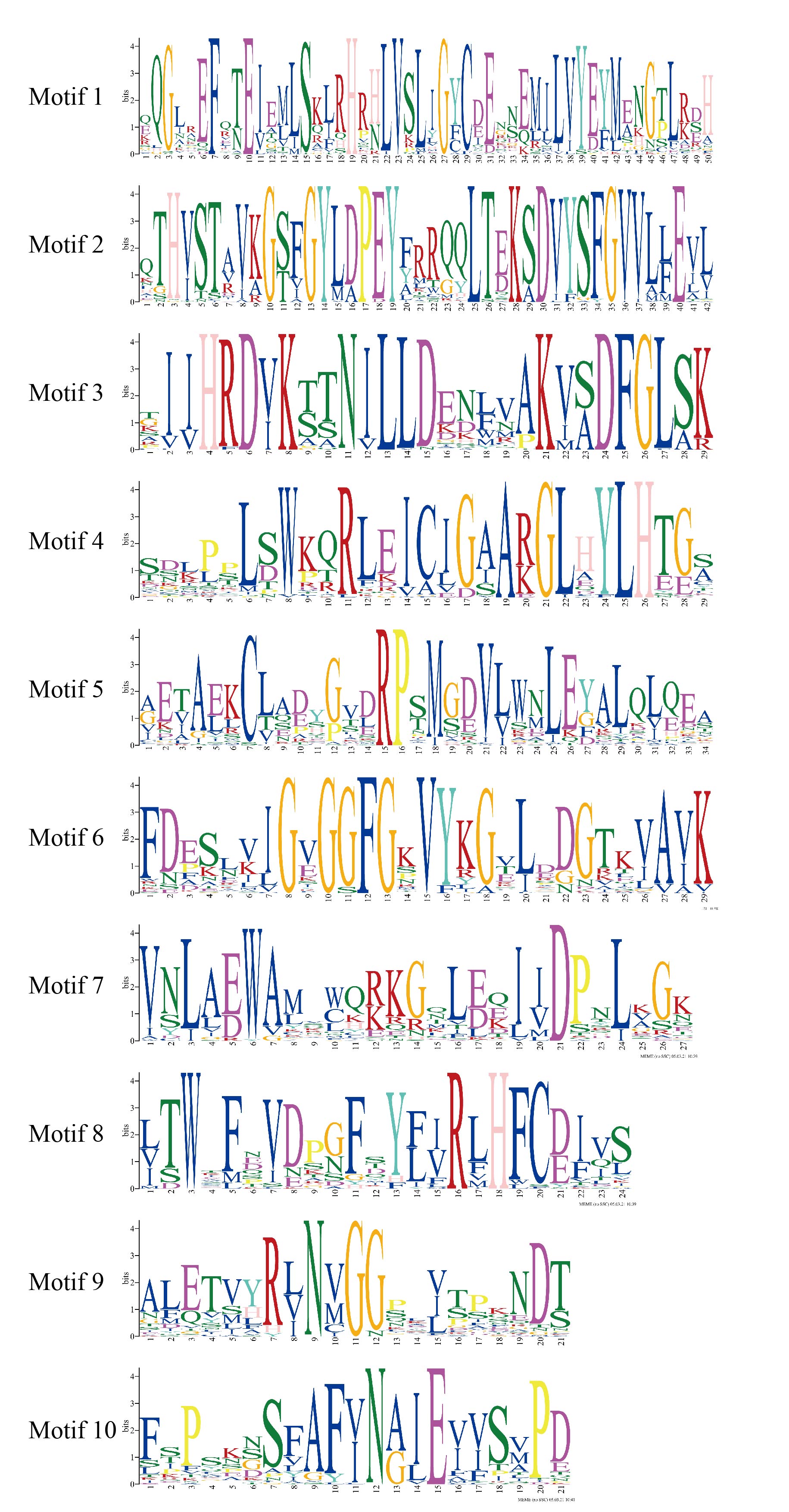

Supplement: Supplementary Table 1 — qRT-PCR primers and sequences used in this study. [file Data_Sheet_1.ZIP › Supplementary_Material/Supplementary Figure 2.jpg]

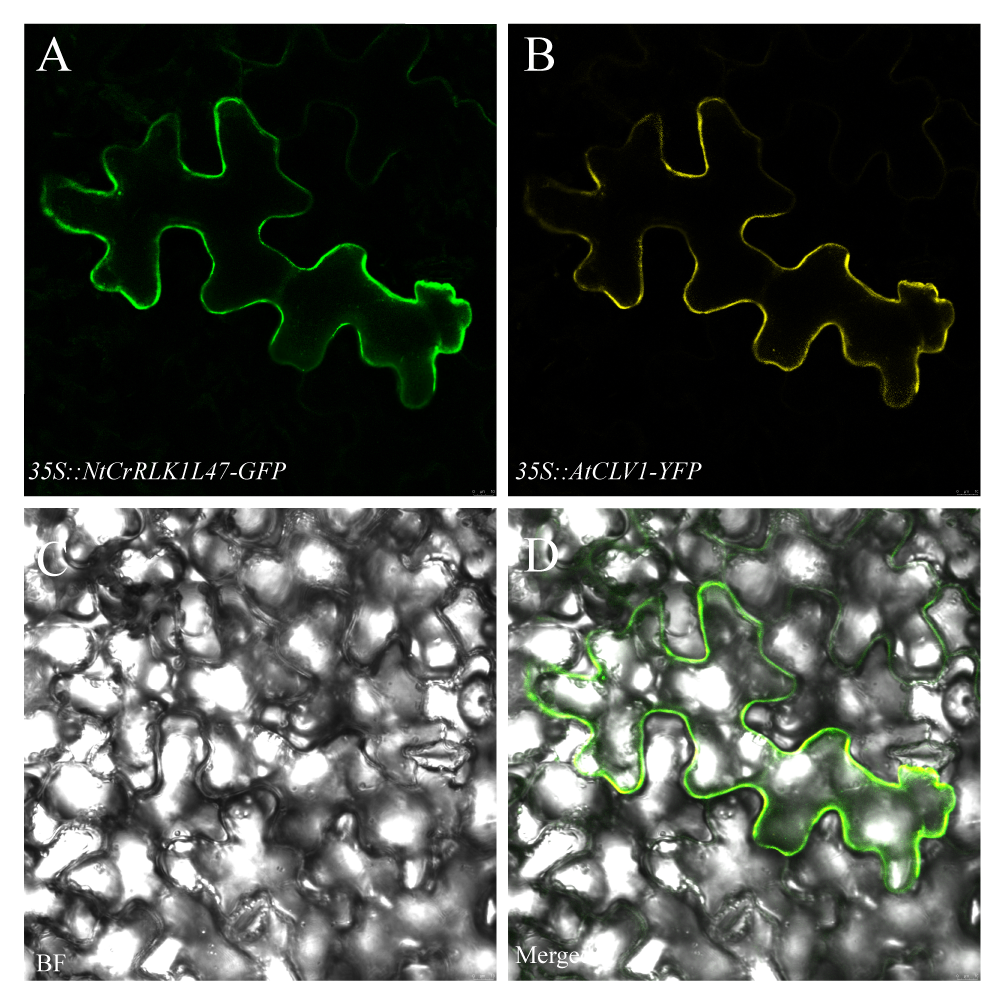

Supplement: Supplementary Table 1 — qRT-PCR primers and sequences used in this study. [file Data_Sheet_1.ZIP › Supplementary_Material/Supplementary Figure 3.tif]

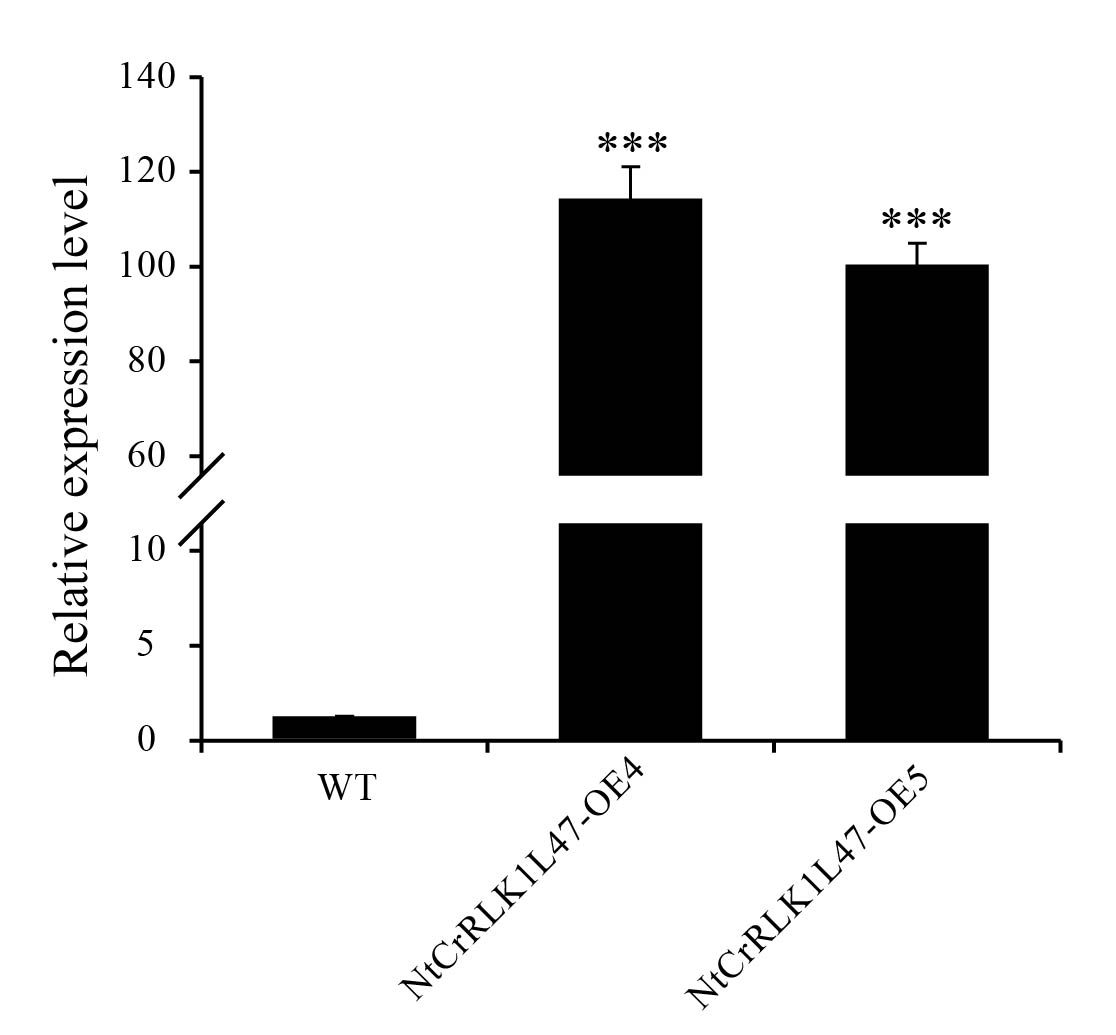

Supplement: Supplementary Table 1 — qRT-PCR primers and sequences used in this study. [file Data_Sheet_1.ZIP › Supplementary_Material/Supplementary Figure 4.jpg]
